# The carnitine system and cancer metabolic plasticity

**DOI:** 10.1038/s41419-018-0313-7

**Published:** 2018-02-14

**Authors:** Mariarosa Anna Beatrice Melone, Anna Valentino, Sabrina Margarucci, Umberto Galderisi, Antonio Giordano, Gianfranco Peluso

**Affiliations:** 1 Department of Medical, Surgical, Neurological, Metabolic Sciences, and Aging, 2nd Division of Neurology, Center for Rare Diseases and InterUniversity Center for Research in Neurosciences, University of Campania “Luigi Vanvitelli”, Naples, Italy; 2 Department of Biology, Sbarro Institute for Cancer Research and Molecular Medicine, Center for Biotechnology, College of Science and Technology, Temple University, Philadelphia, PA USA; 3 Institute of Agro-Environmental and Forest Biology, National Research Council, IBAF-CNR, Naples, Italy; 4 Institute of Bioscience and BioResources, IBBR- CNR, Naples, Italy; 5 Department of Experimental Medicine, Biotechnology and Molecular Biology Section, University of Campania “Luigi Vanvitelli”, Naples, Italy; 6 Department of Medicine, Surgery and Neuroscience, University of Siena, Siena, Italy

## Abstract

Metabolic flexibility describes the ability of cells to respond or adapt its metabolism to support and enable rapid proliferation, continuous growth, and survival in hostile conditions. This dynamic character of the cellular metabolic network appears enhanced in cancer cells, in order to increase the adaptive phenotype and to maintain both viability and uncontrolled proliferation. Cancer cells can reprogram their metabolism to satisfy the energy as well as the biosynthetic intermediate request and to preserve their integrity from the harsh and hypoxic environment. Although several studies now recognize these reprogrammed activities as hallmarks of cancer, it remains unclear which are the pathways involved in regulating metabolic plasticity. Recent findings have suggested that carnitine system (CS) could be considered as a gridlock to finely trigger the metabolic flexibility of cancer cells. Indeed, the components of this system are involved in the bi-directional transport of acyl moieties from cytosol to mitochondria and vice versa, thus playing a fundamental role in tuning the switch between the glucose and fatty acid metabolism. Therefore, the CS regulation, at both enzymatic and epigenetic levels, plays a pivotal role in tumors, suggesting new druggable pathways for prevention and treatment of human cancer.

## Facts

- Malignant cells are capable of creating an equilibrium between producing and consuming energy and metabolic intermediates synthesis to sustain growth and survival.
- Metabolic plasticity makes cancer cells more aggressive and able to metastasize. Oncogenic pathways, nutrient availability, and microenvironment influence cell metabolism.
- The carnitine system is a pivotal mediator in cancer metabolic plasticity, intertwining key pathways, factors, and metabolites that supply an energetic and biosynthetic demand for cancer cells.
- MiRnas and metabolic enzymes regulate metabolic plasticity through the carnitine system suggesting their use for developing new therapeutic strategies.

## Open questions

- What is the role of the carnitine system in cancer metabolism rewiring?
- Is the carnitine system dysregulated in cancerogenesis?
- What is the purpose of epigenetics in the modulation of the expression of proteins belonging to the carnitine system?
- Is it possible to explore new anticancer treatment targeting component(s) of the carnitine system?

Cancer cells must maintain metabolic homeostasis in a wide range of conditions, including harsh microenvironments in which cancer cells must continue to meet the high bio-energetic demand in order to undergo replication^1^. These cells achieve metabolic homeostasis by regulating the dynamics of nutrients present in the microenvironment, and the ability of cancer cells to utilize them to produce energy and to synthesize macromolecules. It is feasible that the capability of cancer cells to employ alternative nutrients in different environments is critical in supporting and affecting their survival. However, tumor cell metabolic plasticity is not just as the result of the metabolic dynamic impact changes induced by the microenvironment and fuel choices of cancer cells. Instead, it is more appropriate to envision metabolic plasticity regarding uptake of alternative metabolic substrates and promotion of metabolic rewiring as a built-in feature that has evolved to allow cancer cells to constantly adapt to changing intracellular- and extracellular metabolic conditions^2^.

Intracellular metabolite concentrations have to fine-tune the signaling networks governing metabolic pathways independently of the environment to ensure a balance between the availability of nutrients and the cellular capacity to use them effectively. Metabolites, through post-translational modifications of metabolite-sensitive protein (i.e. acetylation, methylation or glycosylation), transduce the information on the cell metabolic status, and modulate the activities of signaling proteins, enzymes, and transcriptional regulators^3^. Therefore, it is necessary to understand how a variety of intrinsic and extrinsic factors are integrated to create the metabolic flexibility and to reduce the metabolic dependencies dictated by oncogenic signaling. In this review, we identify the carnitine system (CS) as a gridlock to finely trigger the cancer cells’ metabolic plasticity. In this context, the CS regulation at both the enzyme and the gene level plays a pivotal role in the metabolic flux modulation of tumors, and scientists can target them for therapeutic purposes.

## Nutrients and energy acquisition strategies in cancer cells metabolism

Cancer cells prioritize aerobic glycolysis (Warburg effect), as the primary fuel and convert excess pyruvate to lactate independently from oxygen availability^4^. In addition, glucose is considered to be the primary carbon source that contributes to the production of mitochondrial citrate in cancer cells. The citrate in excess of mitochondrial requirements is exported in the cytosol, converted into acetyl-CoA by ATP-citrate lyase (ACLY), and used for protein acetylation and lipogenesis. While the principles regulating glucose-dependence in cancer cells have been extensively reviewed, we still do not fully understand how cancer cells use many of the metabolic strategies to contribute to core metabolic functions in the presence of nutrients different from glucose. Indeed, in addition to glycolysis, cancer cells can carry out various metabolic strategies such as fatty acid oxidation (FAO)^2,5^. Recent studies have reported that lipids from neighboring adipose tissues, lipoproteins, lysophospholipids, and intracellular storage fat have the potential to maintain viability and growth of cancer cells^6–9^. Fatty acids can satisfactorily fuel cancer cells, since mitochondrial FAO produces much more ATP per mole than oxidation of other nutrients, such as glucose or amino acids. For example, prostate cancer and B-cell lymphomas promote FAO as the main source of energy production and express FAO enzymes at high levels, even under nutrient-replete conditions^10,11^. Again, autophagy and related processes enable tumor growth by sustaining oxidative phosphorylation^12^. Interestingly, the strong dependence of mitochondrial β-oxidation on autophagic and fatty acid (FA) catabolic processes makes some tumors more resistant to nutrient deprivation and environmental stressors^13^.

A peculiarity of several tumors is to present simultaneously two metabolic pathways in opposite directions, such as fatty acid biosynthesis and FAO (“futile cycle”). This paradoxical condition fulfils two fundamental tasks: (a) it provides the biosynthesis of FA important for cancer propagation, while ensuring an important source of ATP and Nicotinamide adenine dinucleotide (NADH) by the catabolism of any FA excess; and (b) it might induce a dynamic switching behavior that is useful in triggering signaling pathway(s) able to overcome metabolic stress maintaining cellular energy homeostasis (i.e. AMP-activated protein kinase; AMPK)^14,15^.

However, unlike glycolytic and lipogenic pathways, where specific metabolic enzymes such as hexokinase 2 and FAS are known to be deregulated by oncogene(s) or by inactivation of tumor suppressors^16,17^, there is limited evidence for cancer-associated abnormal expression or activity of the enzymes directly involved in the FAO pathway. Regarding expression and activity, the knowledge about the regulation of carriers and enzymes that modulate β-oxidation in cancer cells is of extreme interest since their inhibition may significantly affect the tumorigenic potential even in the presence of compensatory metabolic pathways.

## The carnitine system and its implication in cancer cells metabolic plasticity

**Fig. 1:**
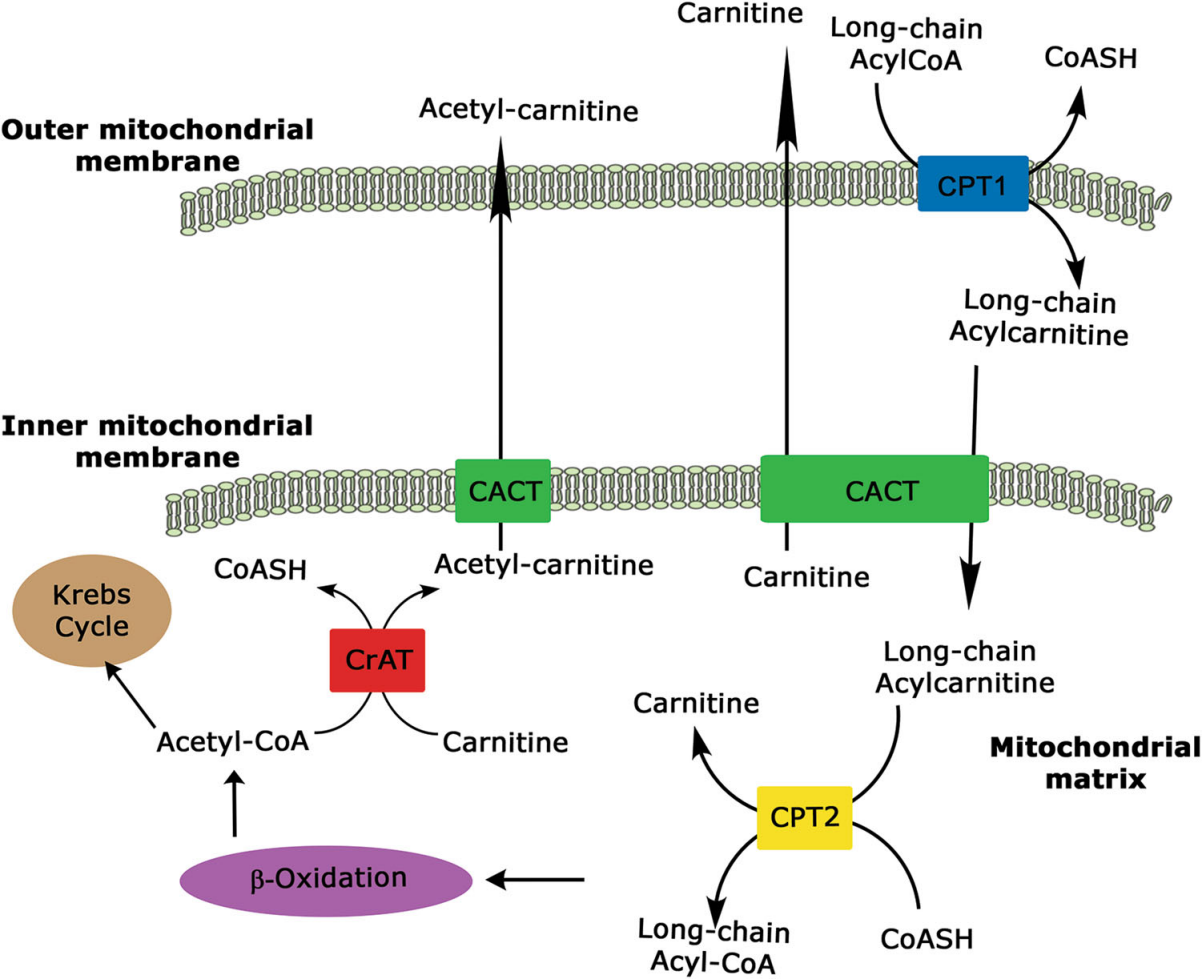
Schematic view of carnitine system. The long-chain fatty acid-CoAs are converted into the carnitine derivatives by CPT1, located on the outer mitochondrial membrane of the contact sites. A specific carnitine-acylcarnitine translocase (CACT) catalyzes the mole-to-mole exchange of carnitine/acetylcarnitine and acylcarnitines promoting the import of the acylcarnitines through the mitochondrial membranes. In the mitochondrial matrix, long-chain acylcarnitines are reconverted to the respective long-chain acyl-CoAs by carnitine-palmitoyltransferase-2 (CPT2) and undergo β-oxidation to produce acetyl-CoAs. Finally, CrAT converts short-chain acetyl-CoAs to their membrane permeant acetylcarnitine counterparts, allowing CACT to export them from mitochondrion to cytoplasm

The use of diet-derived or adipose tissue-released long-chain fatty acids as energy substrates requires about 25 different enzymes and transport proteins, which carry out fatty acids, import, them into mitochondria, and facilitate the β-oxidation steps. In particular, the mitochondrial inner membrane is impermeable to fatty acyl-CoA thioesters and, thus, the specialized CS, for transporting fatty acids across mitochondrial membranes has evolved^18^. Components of the CS are both enzymes able to catalyze the acyl-CoA + carnitine ↔ CoA + acylcarnitines reaction and carrier(s) involved in the bi-directional transport of acyl moieties from cytosol to mitochondria and vice versa. Four components comprise this transporting system: the carnitine palmitoyltransferase 1 (CPT1) and 2 (CPT2), the carnitine-acylcarnitine translocase (CACT), and the carnitine acetyltransferase (CrAT) that close the carnitine cycle, allowing the export of the FAO-produced acetyl-CoA as acetyl-carnitine (Fig. 1).

## Carnitine palmitoyl transferase family

### Carnitine palmitoyltransferase 1A

CPT1A is present mostly in the colon, duodenum, liver, kidney, and small intestine, and its deficiency results in a rare autosomal recessive metabolic disorder of long-chain FAO^19^. The overexpression of CPT1A is often associated with tumor progression in several cancers such as breast, gastric, prostate, lymphoma, leukemia, ovarian, lung, and myeloma^20–23^. Several studies reported that inhibition/depletion of CPT1 leads to apoptosis and suppression of cancer cell proliferation, chemoresistance, and neo-vascularization^24–28^. It has been proposed that CPT1A contributes to cell survival, not only by increasing FAO^29^ but also by stimulating histone acetylase activity in the nucleus^30^. CPT1A can also protect cells from apoptosis by clearing the cytoplasmic long-chain fatty acyl-CoA such as palmitoyl-CoA, and thus impede the production of “palmitate/palmitoyl-CoA/ceramide” involved in the apoptosis pathway activation^31^. Pharmacological inhibition of CPT1A results in impaired cancer cell proliferation in acute myeloid leukemia and intensive cytotoxicity in Burkitt’s lymphoma^23,32^. Interestingly, the CPT1 inhibition decreases β-oxidation, which remarkably attenuates c-myc–mediated lymphomagenesis. This suggests a potential role of CPT1 in the pathogenesis of c-myc-driven cancer. In particular, Ricciardi et al. demonstrated in vitro the anti-leukemic activity of the novel CPT1A reversible inhibitor ST1326 on leukemia cell lines and primary cells obtained from patients with hematologic malignancies, which induces cell growth arrest, mitochondrial damage, and apoptosis^32^. Moreover, Pacilli et al. demonstrated that ST1326 inhibits FAO not only by blocking CPT1A but also by inhibiting CACT activity^23^. In addition, Shao et al. proved that most ovarian cancer cell lines express CPT1A highly and that its inactivation decreased cellular ATP levels, inducing cell cycle arrest at G0/G1^33^. Several studies highlight the role of CPT1A in prostate cancer. Indeed, Schlaepfer et al. showed that the blockage of CPT1A (with etomoxir) and the lipid synthesis/lipolysis (with orlistat) decreased the viability of the androgen-dependent prostate cell lines LNCaP, VCaP, and patient-derived prostate cancer cells^34^. These effects were associated with decreased androgen receptors, mTOR and AKT expression signaling, and increased caspase-3 activation^35^. The upregulated CPT1A expression and activity was also observed in PC3 and LNCaP malignant prostate cells (androgen independent and dependent cells) and human prostate cancer specimens^22^. This recent study identifies an miRNA that targets CPT1A as a potential biomarker for therapy. Upregulated expression of CPT1A has also been determined in breast cancer by an integrated genomic strategy based on the use of gene expression signatures of oncogenic pathway activity^20^. Moreover, Linher-Melville et al. showed that prolactin (PRL) increased the expression and the activity of CPT1A in breast cancer cells with respect to normal cells^29^. The authors suggest that inhibition of FAO may lead to an overall decrease in cancer cell survival, while an increase in CPT1 activity, such as the PRL-mediated response, may provide a supportive environment for malignant cells^36^. Malonyl-CoA, the product of acetyl-CoA carboxylases (ACC), inhibits CPT1A. ACC exists as two related enzymes, the cytosolic isoform ACC1 and the mitochondrion-anchored ACC2. Both enzymes catalyze ATP-dependent carboxylation of acetyl-CoA to form malonyl-CoA. ACC1 is thought to be the predominant isoform mediating fatty acid synthesis, whereas ACC2, the isoform located near CPT1, is the major regulator of CPT1 activity. ACC1 expression has been shown to be frequently upregulated in several tumor types^37^, and have reported the expression and/or activation of ACC2 to be inhibited in a variety of cancers^38^.

### Carnitine palmitoyltransferase 1 B

Carnitine palmitoyltransferase 1 B (CPT1B) is expressed mainly in tissues characterized by high rates of FAO, such as muscle and brown adipose tissue. CPT1B is also expressed in minor quantities in the testis, spleen, duodenum, lymph node, and stomach. Although CPT1A and CPT1B exhibit considerable sequence similarity, the sensitivity of these two enzymes to their physiological inhibitor, malonyl-CoA, differs greatly (CPT1A has a ten-fold higher Ki for malonyl-CoA)^39,40^. CPT1B is involved in human colorectal cancer as determined by Yeh et al.^41^. By using a combined approach of Microarray-Bioinformatic technologies, the authors have revealed the overexpression of CPT1B in clinical tissue specimens of colorectal cancer, demonstrating a potential metabolic mechanism contributing to colorectal cancer. Moreover, recently, Kim et al., have demonstrated the deregulation of some carnitine-acylcarnitine metabolic pathway-associated genes, such as CPT1B, that results in an increased mortality in patients with muscle-invasive bladder cancer^42^.

### Carnitine palmitoyltransferase 1C

The brain-specific carnitine palmitoyltransferase 1C (CPT1C) displays high-affinity to bind malonyl-CoA, but its enzymatic activity cannot be observed using conventional assays^43^. Recent studies showed that CPT1C was localized both in the endoplasmic reticulum and mitochondria, but its presence in endoplasmic reticulum is predominant. CPT1C is involved in cellular energy-sensing pathways and has an important role in the hypothalamic regulation of energy homeostasis^44^. It has been reported that the CPT1C isoform is overexpressed in human tumor cells such as neuroblastoma, several sarcomas of soft-tissues and lung, and malignant peripheral nerve sheath tumors (associated with neurofibromatosis type 1)^45^. When CPT1C is upregulated, it increases FA consumption, and ATP production, facilitating tumor growth and survival^43^. Interestingly, depletion of CPT1C in mice led to delayed tumor development and a striking increase in survival^46,47^. Sanchez-Macedo et al. showed that p53 directly activated CPT1C transcription, conferring to tumor cells resistance^46^. A recent study indicates that CPT1C might be induced in the MCF-7 breast cancer cell line by the indirect action of activated 5′ AMP-activated protein kinase (AMPK)^48^. Moreover, Zaugg et al. showed that MCF-7 breast cancer cells constitutively overexpressing CPT1C displayed an increased FAO-dependent ATP production, and resistance to glucose deprivation or hypoxia^43^. Finally, a recent study revealed that peroxisome proliferator-activated receptor alpha (PPARα) upregulated the expression of CPT1C, in a p-53 independent way, modulating proliferation and senescence of tumor cells^49^. These findings suggest that CPT1C is a regulator of FA homeostasis in cancer cells and might be indirectly involved in the modulation of CPT1A activity through the lowering of cellular malonyl-CoA levels. Indeed, CPT1C binds to malonyl-CoA with the same affinity as CPT1A. Recently, in high-grade glioblastoma, Cirillo et al. showed an association between the expression of CPT1C and ZFP57, a zinc finger protein involved in gene imprinting^50^. Since a truncated form of CPT1C has been demonstrated in the nuclei of human diffuse gliomas, new perspectives are opening on the role of CPT1C expression in cancer cells^51^.

### Carnitine palmitoyltransferase 2

Compared to CPT1 isoforms, less is known about CPT2 deregulation in cancer; nevertheless, a recent study has reported that this enzyme could be considered as an independent prognostic factor in colorectal cancer patients^52^. Another study analyzed the expression of several enzymes involved in FA metabolism in the leukemia cells compared to normal cells. The results showed that leukemia cells presented a higher expression of most CPT isoforms, including CPT2. This suggests that chronic lymphocytic leukemia cells were highly active in de novo FA synthesis as well as in FA catabolism^53^. In addition, a recent paper correlates the phosphorylation state of Src with the expression and activity of CPT1A and CPT2 in triple-negative breast cancer (TNBC)^54^. It is well known that aberrant Src activation plays prominent roles in cancer formation and progression^55^. The authors determined by trans-mitochondrial cybrids and multiple OMICs approaches that TNBC exhibit high levels of ATP through FAO and activates Src oncoprotein through autophosphorylation. The inhibition of FAO by the knocking down of CPT1 and CPT2 significantly decreased the phosphorylation level of Src and the number of metastatic nodules, confirming the role of mitochondrial FAO and CPT genes in Src regulation and their significance in breast cancer metastasis.

## Carnitine translocase and transferase components

### Carnitine/AcylCarnitine translocase

Mitochondria import acylcarnitines through carnitine/acylcarnitine translocase (CACT) located in the inner mitochondrial membrane, which catalyzes a mole-to-mole exchange of carnitines/acetylcarnitines and acylcarnitines^56^. It has been demonstrated that, together with the regulation of CPT1 by malonyl-CoA, acetylation of CACT plays a crucial role in the control of the influx of fatty acyl units into mitochondria^57^. Peluso at al. hypothesized that in obesity-related insulin resistance (IR), skeletal muscle may decrease FAO because of decreased CACT activity^58^. Subsequently another work of the same research team analyzed the metabolic effects of CACT down-expression in human myocyte cells stably transfected with CACT antisense cDNA. This study revealed a complex metabolic situation determined by insulin and palmitate in CACT-deficient cells, and has provided insight into the functional interactions between CACT, CPT1, malonyl-CoA, and acetyl-carnitine^59^. To date, the correlation between CACT and cancer has received little attention, and only a few studies have reported a link between the altered expression of CACT and cancer. Kim et al. have demonstrated that in bladder cancer patients the expression of carnitine enzymes such as CACT has significantly deregulated in tumor tissues compared to normal bladder tissues^42^. Moreover, Valentino et al. demonstrated that in androgen-dependent and -independent prostate tumor cells as well as in human prostate cancer specimens, the overexpression and the increased activity of CACT is a hallmark of prostate cancer^22^.

### Carnitine *O*-acetyltransferase

Carnitine *O*-acetyltransferase (CrAT), located primarily in the mitochondrial matrix, catalyzes the addition or the removal of carnitine from medium- and short-chain acyl-CoA^60,61^, facilitating the efflux of mitochondrial acetyl-CoA to the cytosol and buffering the intracellular pools of acetyl-CoA and carnitine. CrAT deficiency leads to accumulating acetyl-CoA, which exerts an allosteric inhibiting effect on pyruvate dehydrogenase (PDH), a rate-limiting enzyme for pyruvate entry into the Krebs cycle. This metabolic alteration has been associated with metabolic diseases or to insulin deficiency^62–64^. Interestingly, the CrAT activity is reduced during obesity and aging, leading to impaired glycemic control^65^. Studies in Crat knockout mice demonstrated that CrAT deficiency leads to abnormal fuel selection, which results in a perturbation of glucose homeostasis and suggest that deficits in CrAT activity might contribute to diet-induced metabolic inflexibility by exacerbating the Randle glucose-fatty acid cycle^66^. The higher CrAT amount determined both in prostate cancer cells (PC3 and LNCaP, androgen-dependent and androgen-independent, respectively) and prostate tumor biopsy by Valentino et al. highlight the importance of CrAT to contribute to maintaining a high metabolic plasticity of prostate cancers^22^. Finally, CrAT might play a fundamental role in histone acetylation in cancer cells. From a canonical point of view, ATP Citrate Lyase (ACL) produces acetyl-CoA from mitochondrion-derived citrate. Most of the acetyl-CoA produced by ACL originally derives from glucose or glutamine carbon. In addition to contributing to FA synthesis, acetyl-CoA can regulate cell growth by controlling the expression of genes involved in this process by histone acetylation. Alternatively, acetylcarnitine, produced in excess in mitochondria by CrAT, is transported into cytosol by CACT and enters the nucleus, where a nuclear CrAT converts the acetylcarnitine to acetyl-CoA^67^, and it becomes a source of acetyl groups for histone acetylation. Therefore, besides citrate-derived acetyl-CoA by nuclear ATP-citrate lyase^68^, the carnitine-mediated supply of acetyl groups is also an important source of acetyl-CoA for nuclear histone acetylation. Indeed, genetic deficiency of the translocase markedly reduced the mitochondrial acetylcarnitine-dependent nuclear histone acetylation, indicating the significance of the carnitine-dependent mitochondrial acetyl group contribution to histone acetylation.

## Metabolic intermediates and carnitine system

Metabolic pathways are monitored and controlled by allosteric or post-translational regulation of enzymes that catalyze specific biochemical reactions. Afterwards, alternative splicing, mRNA stability, translation, and protein degradation control the abundance of enzymes (“long-term regulation”)^69^. In this view, nutrients, such as glucose or free fatty acids, provide intermediate metabolites the ability to interact directly with an enzyme (the metabolic sensor) that rules the rate limiting step of a metabolic process, thus regulating substrate preference. A case in point is the so-called “glucose-fatty acid cycle”, a prime example of tightly coordinated cellular energy metabolism that provides a mechanistic reciprocal regulation of lipid and glucose oxidation to maintain cell homoeostasis and to avoid mitochondrial overloading. Interestingly, β-oxidation and aerobic glycolysis are connected to each other through cross-signaling in such a manner that beta-oxidation not only is compatible with ongoing aerobic glycolysis, but also it promotes the Warburg Effect. Indeed, the PDH complex, which decarboxylates glycolytically derived pyruvate to acetyl coenzyme A and links cytoplasmic glycolysis to the mitochondrial tricarboxylic acid (TCA) cycle, is modulated by reversible phosphorylation by PDH kinase (PDK). Nicotinamide adenine dinucleotide (NADH) and acetyl-CoA, two metabolic intermediates produced in the course of FAO, induce activation of PDK, which in turn phospho-inactivates the E1α subunit of the PDH complex, leading to lower rates of glucose oxidation and higher rates of lactate release^70^. Interestingly, multiple transcription factors, such as Myc, Wnt, and hypoxia inducible factors, can cause a transcriptional increase of one or more PDK isoforms in a cancer cell. A cross-talk between metabolic intermediates produced by aerobic glycolysis, and enzymes belonging to the carnitine cycle is also present in cancer cells. The relationship between lactate and cancer growth reflects the pleotropic actions of this molecule able to influence the metabolic phenotype of the cancer cells. A recent study has demonstrated that lactate-induced acidification of the microenvironment over a period of weeks induces a metabolic adaptation of the tumor cell population, promoting β-oxidation as a metabolic strategy (the Corbet−Feron Effect)^71^. In addition, cancer cells chronically exposed to an acidic pH reveal a downregulation of the mitochondrial ACC2 isoform, the enzyme that regulates CPT1 activity by malonyl-CoA synthesis. Therefore, only ACC1 isoform is expressed in tumor cells under acidosis to produce malonyl-CoA as a substrate for FA synthesis. Of interest, the down-expression of ACC2 results from an epigenetic process linked to histone deacetylation at the ACACB promoter by Sirtuin 1/6 that are activated by high cytosolic NAD^+^ levels associated with enhanced FAO and reduced glucose metabolism.

The non-enzymatic mitochondrial protein hyperacetylation induced by the increased availability of mitochondrial acetyl-CoA avoids the risk associated with mitochondria overfeeding, restraining the activity of the respiratory complex I and modulating CACT activity. Indeed, it has been demonstrated that acetylation plays a key role in the control of CACT function that, together with the long-chain acyl-CoA dehydrogenase, contributes to β-oxidation regulation. CACT acetylation compromises its activity, causing a decrease in its transport function. This mechanism represents a control of the influx of fatty acyl units into mitochondria in response to the intra-mitochondrial acetyl-CoA level, in addition to the finely regulated mechanism of CPT1 by malonyl-CoA. CACT acetylation mechanism is in line with a dynamic post-translational protein control that could undergo an on/off switch induced by deacetylation/acetylation linked to the availability of acetyl-CoA^57^.

Recently, it was found that FA-derived acetyl-CoA is a significant font of carbon for acetylation of certain histone lysine, even in the presence of glucose^72^. Again, CS might be fundamental not only to produce but also to transport acetyl-CoA from the mitochondria to the nucleus for supplying acetyl-CoA for histone acetylation. Acetyl-carnitine synthetized in mitochondria by CrAT is transported out mitochondria by CACT and transformed back to acetyl-CoA by CrAT present in the nucleus. In this model CS assumes a mechanistic role by which FA are perceived and integrated into the epigenome.

**Fig. 2:**
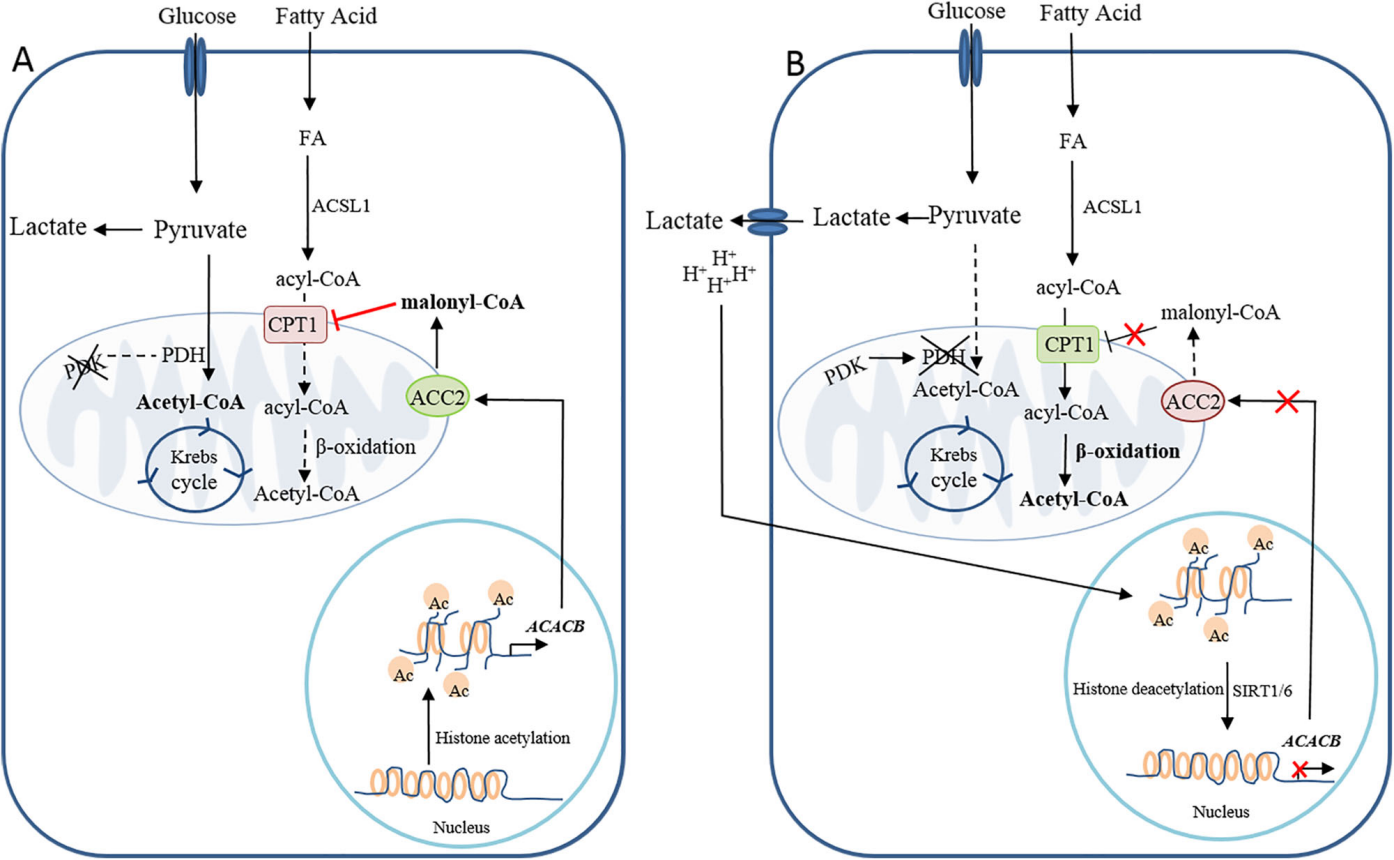
Epigenetic ACC2 control modulates the reprogramming of fatty acid metabolism in cancer cells. **a** Epigenetic control of *ACACB* by histone acetylation induces an increased expression of both ACC2 and its catalytic product, malonyl-CoA, leading to the inhibition of CPT1A activity. On the contrary, **b** histone deacetylation by sirtuin(s) (SIRT1/6), also promoted by lactate-induced acidification of the microenvironment, leads to a decreased expression of *ACACB* and consequently enhances CPT1A activity. NADH and acetyl-CoA, two metabolic intermediates produced in the course of FAO, promote activation of PDK, which in turn phospho-inactivates the E1α subunit of the PDH complex, leading to lower rates of glucose oxidation and higher rates of lactate release

**Fig. 3:**
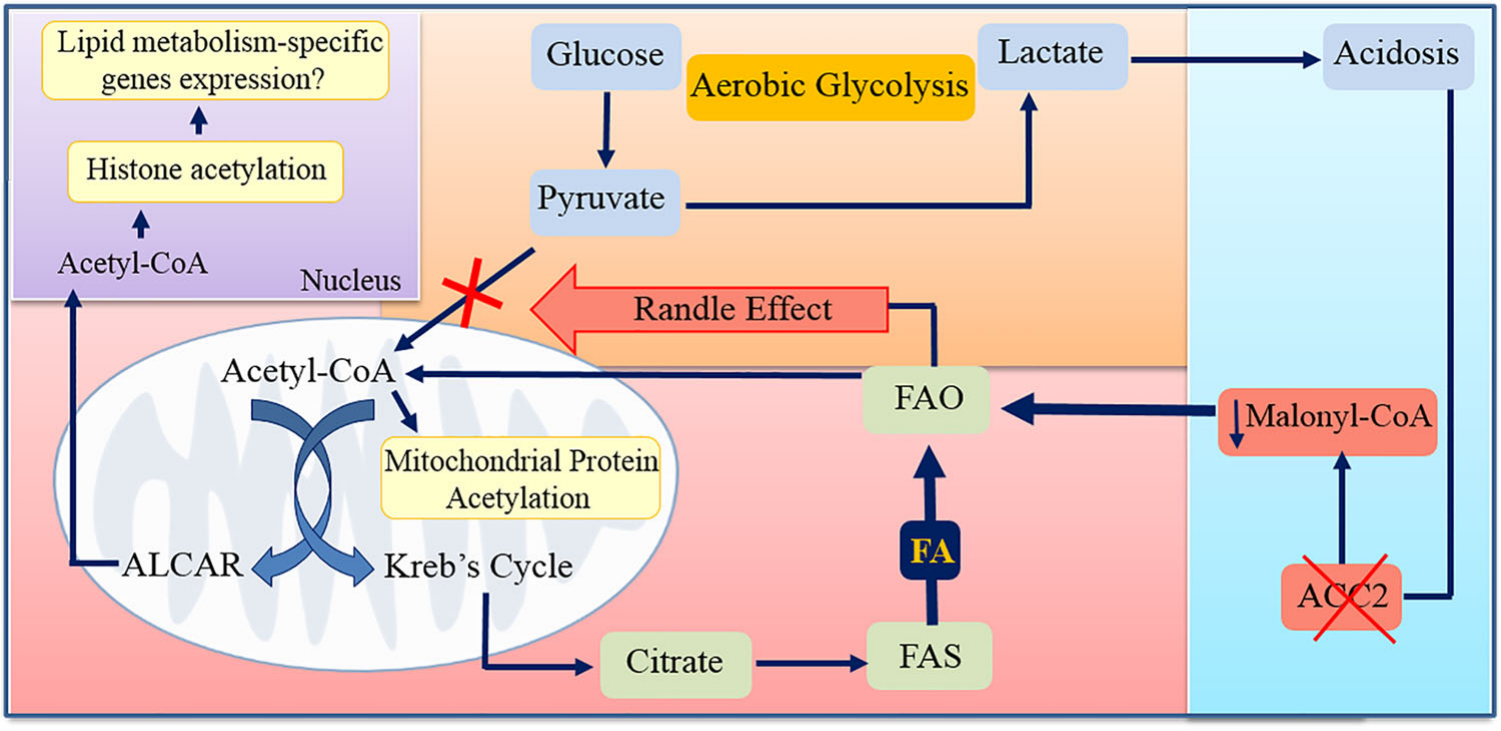
The involvement of carnitine cycle in cancer cell metabolism. In cancer metabolism the aerobic glycolysis induces the conversion of the pyruvate (the end product of the glycolysis) into lactate (shown in orange) leading to the acidification of the microenvironment. The increased acidosis (shown in blue), in association with epigenetic mechanism(s), promotes the downregulation of the mitochondrial ACC2 isoform that in turn increases FAO (shown in red) via CPT1A upregulation. NADH and acetyl-CoA, produced in excess during FAO, promote the conversion of pyruvate into lactate through the inhibition of PDH by PDK (Randle effect). The increased availability of mitochondrial acetyl-CoA enhances the intra-mitochondrial non-enzymatic acetylation of proteins both of the Kreb’s cycle and of the carnitine cycle, avoiding the risk associated with mitochondria overfeeding. In addition, the excess of acetyl-CoA is exported in the cytosol either as citrate or as acetyl l-carnitine (ALCAR). The citrate is converted into acetyl-CoA by ACLY for the synthesis of fatty acids that might be re-imported into the mitochondria for beta-oxidation (Futile Cycle). ALCAR, shuttled to the nucleus (shown in violet), can be used as source of acetyl groups for histone acetylation, probably contributing to lipid metabolism-specific gene expression

All these considerations are schematized in Figs. 2 and 3.

## Small-non-coding RNA (miRNAs) as a carnitine system regulator

**Fig. 4:**
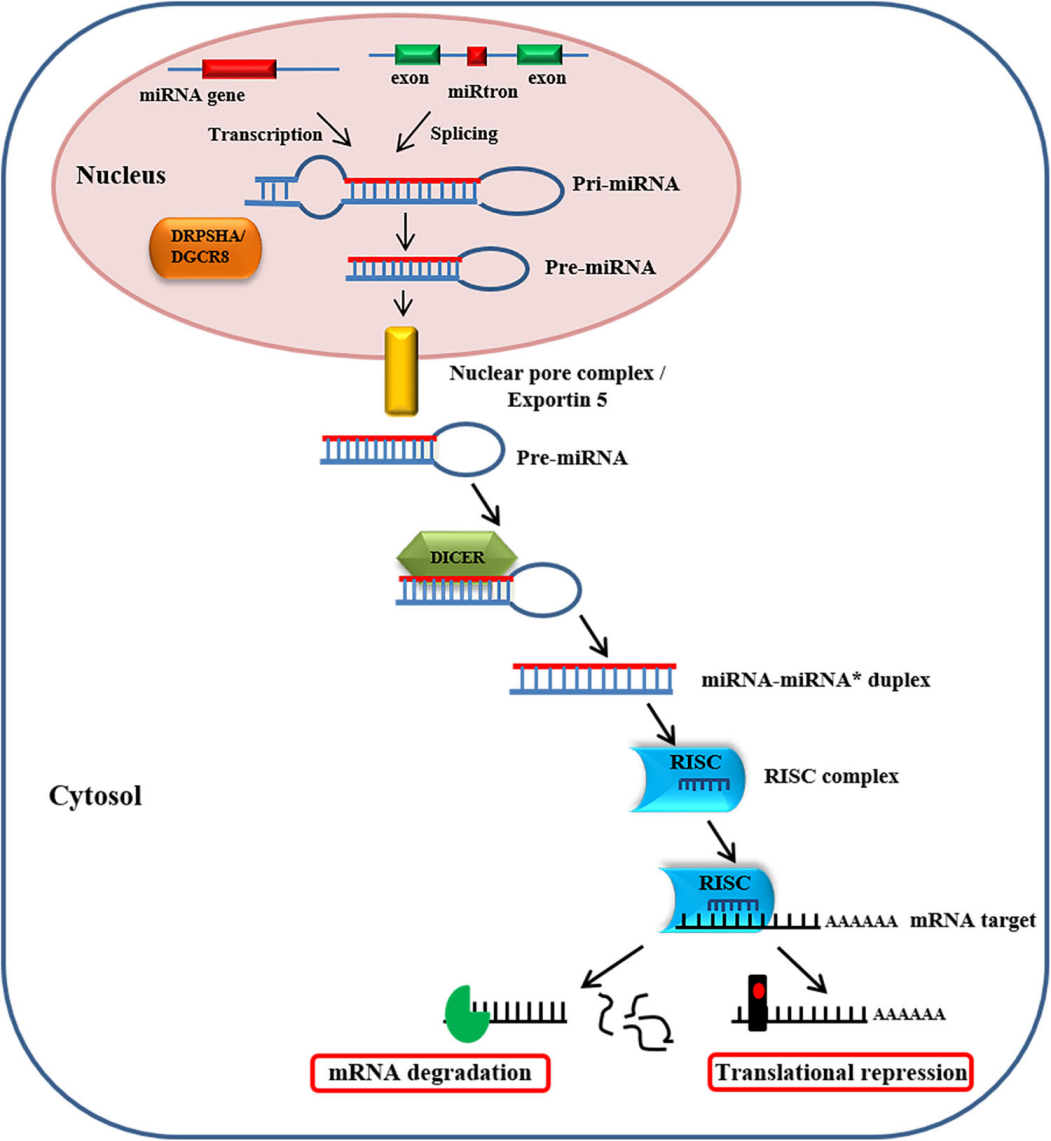
miRNAs biogenesis and mechanism of action. MicroRNAs are transcribed by RNA polymerases II and III in pri-miRNAs, generating precursors that undergo a series of cleavage events to form mature microRNA. Drosha, the first nuclear ribonuclease III, recognizes pri-miRNA and cuts the double-stranded RNA freeing a pre-miRNA. Pre-miRNA hairpin is exported from the nucleus in a process involving the nucleocytoplasmic protein Exportin-5. In the cytoplasm, the pre-miRNA hairpin is cleaved by the RNase III enzyme Dicer in an miRNA duplex of 18–22 nt. Although either the duplex strands may potentially act as functional miRNAs, only one strand is usually incorporated into the RNA-induced silencing complex (RISC) where the miRNA and its mRNA target interact. Mature miRNA acts either by degrading the mRNA target or by inhibiting its translation

Among all small non-coding RNAs, microRNAs (miRNAs) (Fig. 4), 18–25 nucleotides in length, are well characterized as post-transcriptional regulators of mRNAs^73,74^ and are considered a potential source of biomarkers for many diseases, including cancer^75^.

MiRNAs play important regulatory roles in a variety of biological processes such as cell proliferation, intercellular signaling, cell growth, cell death, cellular differentiation, apoptosis, and cancer metabolism^76–78^. Their expression profiles have been found to be tissue type-specific and frequently deregulated in various cancers^79^. MiRNAs have unique characteristics (i.e. stability, tissue specificity, easily detected and manipulated) that make them potential therapeutic targets for cancer treatment^80^. Emerging evidence demonstrates that miRNAs are critical regulators of lipid synthesis and FAO^81^ resulting in defective cell metabolism and carcinogenesis^82^ directly targeting key enzymes or transcription factors as oncogenes and tumor suppressors^81^ as shown in Table 1.

**Table 1:** miRNAs involved in cancer metabolic plasticity

MiRNAs	Target	Reference
miR-122	Cholesterol biosynthesis	^88–90^
miR-370	Fatty acid oxidation, CPT1A	^91^
miR-378/378*	Lipid metabolism, CrAT	^92, 93^
miR-335	Lipid metabolism and adipogenesis	^94^
miR-205	Lipid metabolism	^95^
miR-143	Adipocyte differentiation	^96^
miR-27	Adipolysis	^97^
miR-33a/b	Cholesterol efflux and β-oxidation	^98–100^
miR-185	Lipogenesis and cholesterogenesis	^101^
miR-342	Lipogenesis and cholesterogenesis	^101^
miR-124	CPT1A	^27^
miR-129	CACT	^27, 102^

**Fig. 5:**
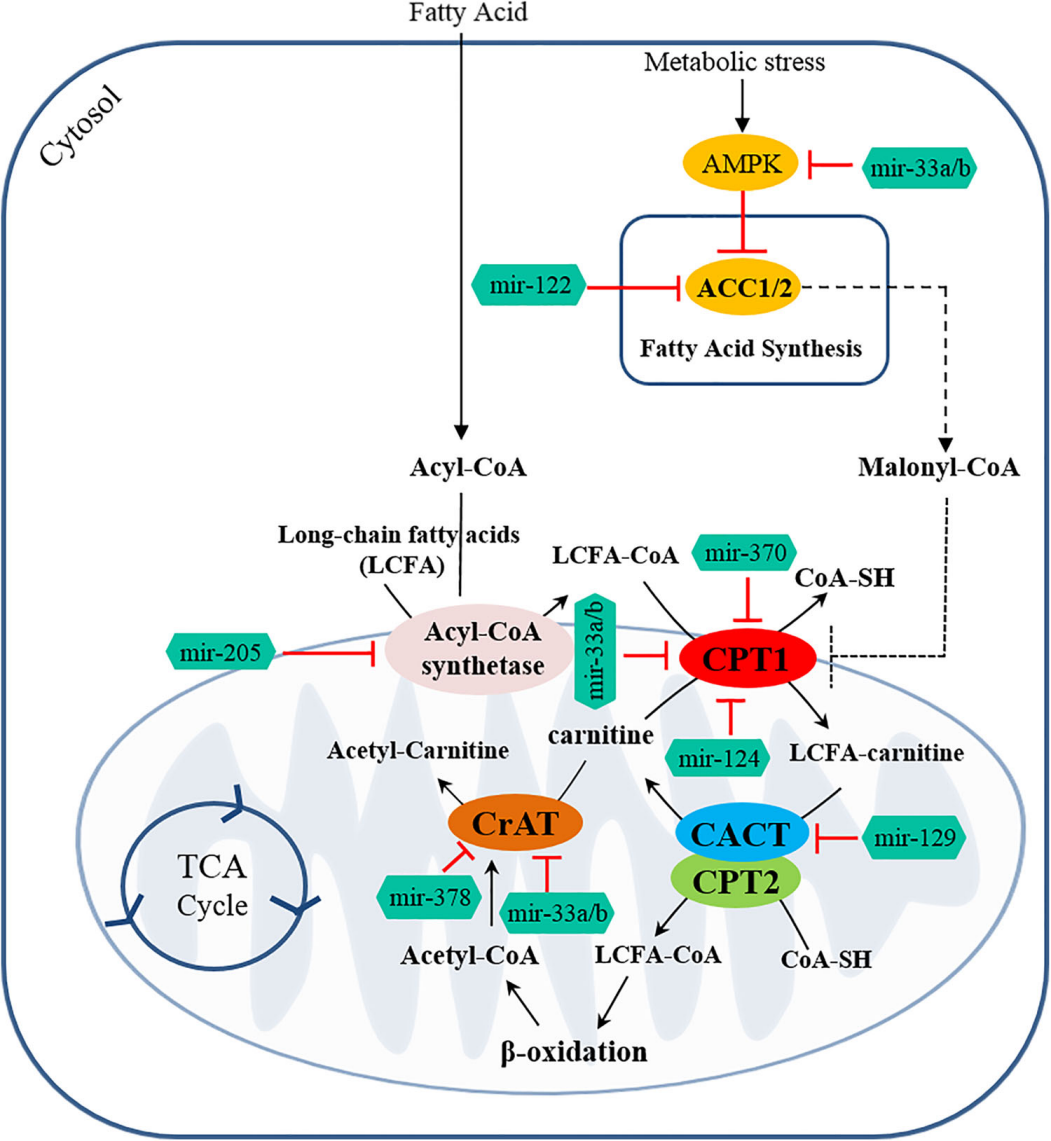
MiRNAs influence the carnitine system components. MiRNAs regulate cell metabolic plasticity by modulating the expression of enzymes involved in several metabolic pathways. MiRNAs affect both directly and indirectly the carnitine system components. Mir-33a/b and miR-122 target AMPK (activated by metabolic stress) and ACC1/2 respectively, whereas miR-205 targets the acyl-CoA synthetase, indirectly regulating the components of carnitine system. In addition, the carnitine system components are directly regulated by miR-370, miR-124 (CPT1A), miR-129 (CACT), miR-33a/b (CPT1A and CrAT), and miR-378 (CrAT)

MiR-122 was the first miRNA identified as tissue-specific, and it is the most abundant in liver involved in lipid metabolic reprogramming^83^. MiR-122 inhibition leads to decreased plasma cholesterol and triglyceride levels associated with altered lipids biosynthesis and increased FAO. Several genes involved in fatty acid synthesis and oxidation were altered in mice treated with anti-miR-122 including FAS, ACC1, and ACC2^84^. Furthermore, miR-122 silencing in high-fat-fed mice reduced hepatic steatosis, with a decrease in cholesterol synthesis and stimulation of FAO^85^. Recently, Iliopoulos et al. identified a new miRNA, miR-370, that has effects on lipid metabolism similar to miR-122. MiR-370 targets CPT1A reducing FAO. Particularly, the human hepatic cell line HepG2 transfection with miR-370 upregulates the expression of miR-122. This upregulation leads to an increased expression of lipogenic genes, including sterol regulatory element-binding proteins (SREBP1c) and diacylglycerol acyltransferase-2 (DGAT2), which suggests that miR-370 provides a further point of regulation of this pathway^86^. Interestingly, components of cholesterol efflux and fatty acid metabolism are regulated by miR-33a and miR-33b. These miRNAs reside in intronic regions within SREBP-1 and 2, the key transcriptional regulators of lipid metabolism^87^, and control the expression of carnitine *O*-octanoyltransferase (CROT), CPT1A, hydroxyacyl coenzyme A (hydroxyacyl-CoA) dehydrogenase subunit beta (HADHB), and AMPK, targeting their 3′ UTR^88^. Another important miRNA regulating cell metabolism is miR-378/378*, embedded within gene encoding peroxisome proliferator-activated receptor gamma coactivator 1-beta (PGC-1β), a transcriptional regulator of oxidative energy metabolism. In breast cancer cells, a high level of miR-378* induces the metabolic shift from an oxidative to a glycolytic bioenergetics pathway by inhibiting the expression of two PGC-1β partners, ERRγ (estrogen-related receptor gamma) and GABPA (GA binding protein transcription factor, alpha subunit). This leads to a reduction in TCA cycle gene expression and oxygen consumption as well as an increase in lactate production and cell proliferation^89^. Targets of miR-378 are also CRAT; indeed, it has been shown that mice genetically lacking miR-378 and miR-378* are resistant to high-fat-diet-induced obesity and display enhanced mitochondrial FA metabolism and elevated oxidative capacity of insulin-target tissue^90^. Moreover, Valentino et al. have demonstrated that the downregulation of hsa-miR-124-3p, hsa-miR-129-5p, and hsa-miR-378 induces an increase in both expression and activity of CPT1A, CACT, and CrAT in malignant prostate cells^22^. In addition, the analysis of human prostate cancer and prostate control specimens confirmed the aberrant expression of miR-124-3p, miR-129-5p, and miR-378 in primary tumors. Forced expression of the above-mentioned miRNAs affected tumorigenic properties, (i.e. proliferation, migration, and invasion), in PC3 and LNCaP cells regardless of their hormone sensitivity. MiR-143, miR-27, miR-335, miR-14, and miR-205 have been recently associated with lipid metabolism and adipocyte differentiation^91^. MiR-27a inhibits the expression of several lipid metabolic genes, including SREBP1-2, FASN, and PPARα/γ, by reducing lipid synthesis and increasing lipid secretion from cells^92^. The expression of MiR-335 is modulated by lipid loading, resulting in overexpression in liver and adipose tissue of obese mice^93^. However, the role of miR-335 in triggering lipid metabolism still remains unknown. Another miRNA implicated in the regulation of lipid metabolism is miR-14. Zu et al., indeed, demonstrated in *Drosophila melanogaster* that the deletion of miR-14 increased the levels of triacylglycerol and diacylglycerol while its overexpression resulted in the converse effect^94^. Recently, it has been found that miR-205 deregulates lipid metabolism in hepatocellular carcinoma by targeting acyl-CoA synthetase ACSL1, a lipid metabolism enzyme in liver^95^. Furthermore, in prostate cancer cells, miR-185 and miR-342 control lipogenesis and cholesterogenesis by reducing the expression of SREBP-1/2 and downregulating their targeted genes, including fatty acid synthase^96^. The function of miRNAs on lipid metabolic plasticity reveals molecular strategies aimed to control metabolic flux by miRNAs in cancerogenesis, thus lighting up one of miRNAs therapeutic aspect^97^ (Fig. 5).

## Concluding remarks

Cancer metabolic plasticity allows tumor cells to survive in the face of adverse environmental conditions. Metabolites involved in metabolic plasticity must be able to fluctuate in response to internal or external perturbations. Therefore, identifying regulatory nodes within the metabolic network is challenging due to its complex structure. Although today several studies have investigated the mechanism involved in metabolic plasticity, but key pathway has not been identified which could be considered a unique target for cancer therapy. We critically review how cancer cells control the metabolic regulatory mechanisms. Therefore, we have emphasized the crucial role of the CS and its fine-tuned regulation, which is both enzymatic- and miRNA-dependent, as the primary factor in metabolic cancer flexibility.
